# Definition and characterization of localised meningitis epidemics in Burkina Faso: a longitudinal retrospective study

**DOI:** 10.1186/1471-2334-12-2

**Published:** 2012-01-05

**Authors:** Haoua Tall, Stéphane Hugonnet, Philippe Donnen, Michèle Dramaix-Wilmet, Ludovic Kambou, Frank Drabo, Judith E Mueller

**Affiliations:** 1 Agence de Médecine Préventive, 164 rue de Vaugirard, 75015 Paris, France; 2 World Health Organization, Department of Epidemic and Pandemic Alert and Response (HSE/EPR), 20 Avenue Appia, 1211 Geneva 27, Switzerland; 3 Université Libre de Bruxelles, Route de Lennik 808, Bruxelles 1070, Belgique; 4 Ministry of Health, 03 BP 7035, Ouagadougou, Burkina Faso

## Abstract

**Background:**

The epidemiology of meningococcal meningitis in the African meningitis belt is characterised by seasonality, localised epidemics and epidemic waves. To facilitate research and surveillance, we aimed to develop a definition for localised epidemics to be used in real-time surveillance based on weekly case reports at the health centre level.

**Methods:**

We used national routine surveillance data on suspected meningitis from January 2004 to December 2008 in six health districts in western and central Burkina Faso. We evaluated eight thresholds composed of weekly incidence rates at health centre level for their performance in predicting annual incidences of 0.4%and 0.8% in health centre areas. The eventually chosen definition was used to describe the spatiotemporal epidemiology and size of localised meningitis epidemics during the included district years.

**Results:**

Among eight weekly thresholds evaluated, a weekly incidence rate of 75 cases per 100,000 inhabitants during at least two consecutive weeks with at least 5 cases per week had 100% sensitivity and 98% specificity for predicting an annual incidence of at least 0.8% in health centres. Using this definition, localised epidemics were identified in all but one years during 2004-2008, concerned less than 10% of the districts' population and often were geographically dispersed. Where sufficient laboratory data were available, localised epidemics were exclusively due to meningococci.

**Conclusions:**

This definition of localised epidemics a the health centre level will be useful for risk factor and modelling studies to understand the meningitis belt phenomenon and help documenting vaccine impact against epidemic meningitis where no widespread laboratory surveillance exists for quantifying disease reduction after vaccination.

## Background

Most episodes of epidemic meningitis occur in the African Meningitis Belt and have been caused by serogroup A of *Neisseria meningitidis *(Nm) [1], although serogroups W135 and X have been found to cause epidemics during the last decade [2-5]. Apart from the current introduction of serogroup A meningococcal conjugate vaccine in Mali, Burkina Faso and Niger, three core countries of the meningitis belt, emergency mass vaccination campaigns with polysaccharide serogroup A/C or A/C/W135 vaccines are organized for epidemic response. This response is launched as soon as weekly incidence rates, usually at the district level, meet the currently recommended threshold for epidemic response [6,7] of 10 or 15 cases/100,000 per week for populations ≥ 30,000 inhabitants (threshold depending on vaccination status and the epidemiology of previous years) and 5 cases per week or doubling of cases over three weeks for populations < 30,000 inhabitants. These thresholds have been developed to predict the occurrence of an epidemic, previously defined at the district level as an annual incidence of 0.1% [7], and are used to launch reactive vaccination campaigns.

To this purpose, all health centres in the countries above report on a weekly basis suspected cases of bacterial meningitis to the sanitary districts, where data are aggregated, analysed and sent to the national level [8]. Furthermore, district level data aggregates are also used for studies of disease trends, risk factor and modelling studies which aim to understand and predict the occurrence of meningitis epidemics [9]. The exclusive use of district level aggregates may be problematic as meningitis epidemics often occur in a highly localised manner and may concern only a few villages, as reported by some studies [10-12]. For instance, when a district declares an epidemic, high incidence rates in a few health centres often are responsible for the increased average incidence, while epidemics in small communities may not be recognized on the basis of district-level data. We recently proposed a model [13] according to which localised epidemics are the basic unit of epidemic meningococcal disease and occasionally form epidemic waves that span larger regions if additional factors lead to a simultaneous widespread occurrence. However, little is known about the sub-district epidemiology of epidemic meningitis, for which evidence has anecdotic rather than quantitative character. One major reason for this is the absence of a definition for localised epidemics on the health centre level. In the present study, we aimed to establish a definition of localised meningitis epidemics in the meningitis belt based on health centre data and to use this definition to describe the spatiotemporal epidemiology of localised epidemics in Burkina Faso over half a decade.

## Methods

We used routine meningitis surveillance data from six sanitary districts in Burkina Faso, selected by convenience of existing public health collaboration. The Hauts-Bassins sanitary region (1,608,900 inhabitants in 2007) consisted of five districts (Secteur 15, Secteur 22, Dandé, Houndé and Orodara sanitary districts) with 173 health centres. Boulsa sanitary district (302,600 inhabitants in 2007) is located in the Centre Nord sanitary region and comprised 28 local health centres. Where the sanitary map changed during the observed period, we assigned health centres to districts according to the older map. Both regions experienced epidemic waves of meningitis predominantly due to NmA during 1996-1998 and 2006-2008, and predominantly due to NmW135 during 2002-2003 [14**]**. Reactive mass campaigns of meningococcal serogroup A/C polysaccharide vaccine were conducted in all districts during 2008, with the exception of Dandé and Houndé and some health centres in eastern and southern Secteur 15, which were vaccinated already during 2006 (information: Ministry of Health, Burkina Faso).

On a weekly basis, the statistical units of sanitary districts in Burkina Faso collect case reports of suspected meningitis from health centres and aggregate them at the district level. The case definition is based on clinical criteria such as fever and meningeal signs without consideration of laboratory confirmation [15]. Although data are usually presented for the whole district, the weekly health centre data are stored in specific electronic files, which we collected to create a database including reports for weeks 1/2004 to 52/2008 in Secteur 15, Dandé and Houndé, weeks 1/2005 to 52/2008 in Secteur 22 and Orodara (all Hauts-Bassins region) and weeks 1/2006 to 15/2008 in Boulsa (Centre Nord region). Data before 2004 usually were missing or on paper files and therefore not included. Weekly reports from the Bobo-Dioulasso university hospital and district hospitals were excluded from analyses, as it was not possible to affiliate residency of patients (N = 813) to specific health centres.

Population data for the calculation of incidence estimates were drawn from the 1996 national census and updated by applying an estimated annual growth rate of 3% for rural areas and 6% for urban areas [16]. Sixty-two percent of included health centre areas had < 10,000 and 3% > 30,000 inhabitants. In Burkina Faso, 87% of the population live within 10 km of a health centre [17].

For descriptive analyses, we calculated weekly incidence rates (WIR) as weekly number of cases per 100,000 inhabitants and annual incidences (%) as annual number of cases per 100 inhabitants. We used Poisson models to test the variability of annual incidences within district years (Wald test) and to test the difference of annual incidences between health centres with and without localised epidemics. We chose as the primary reference an annual incidence above the 95th percentile of annual incidences in all health centre years in our data base, which was 0.4%. The secondary reference corresponded to the incidence reported during a focal epidemic in Burkina Faso during 2006, which reached 0.8% in one health centre [11]. We then formulated and tested eight thresholds of WIR at the health centre level to predict the occurrence of a localised epidemic: The first threshold was the recommended threshold for epidemic response in populations with < 30,000 inhabitants, applied to health centre level data (RT@HC) [6]. The other seven thresholds were WIRs of ≥ 25, 50, 75, 100, 150, 200 or 250 cases per 100,000 during at least two consecutive weeks and ≥ 5 cases reported during at least one of the two weeks. We added the requirement of two consecutive weeks and the minimum number of cases to limit bias due to operational issues such as non-continuous reporting or incidence estimates with wide confidence intervals in small populations. These thresholds will be referred to as LEs. For example, LE25 identified health centres with WIR of ≥ 25 during at least two consecutive weeks and at least five cases reported during at least one. The eight thresholds were evaluated for their sensitivity and specificity in predicting health centres with eventual annual incidence of ≥ 0.4% and ≥ 0.8%. The threshold with the optimised diagnostic performance was chosen as the definition of localised epidemics and was used to describe the spatiotemporal epidemiology of localised epidemics in the six districts during 2004-2008. The duration of localised epidemics included the two weeks required by the definition. Using Spearman's correlation, we evaluated the hypotheses of correlations between district level annual incidence and characteristics of the localised epidemics in the district, such as number of localised epidemics, size of the health centre area population, median peak WIR and median annual incidence in localised epidemics.

All analyses were performed on Stata/IC10 software (StataCorp Texas USA). We did not use any information that could allow an individual patient identification, therefore no ethics committee approval was sought for this research, for which we obtained authorization by the Ministry of Health of Burkina Faso.

## Results

**Figure 1:**
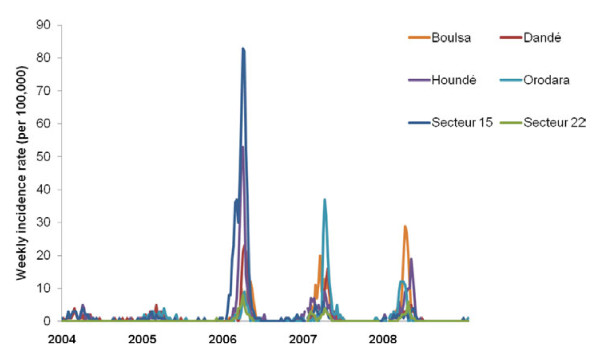
**Weekly incidence rates of suspected meningitis in six sanitary districts of Burkina Faso, 2004-2008**. The districts are situated in the sanitary regions Hauts-Bassins (Dandé, Houndé, Orodara, Secteur 15 and Secteur 22) and Centre Nord (Boulsa). The weekly incidence rates (per 100,000 inhabitants) are based on the national routine case reporting system and population estimates as provided by sanitary authorities.

**Figure 2:**
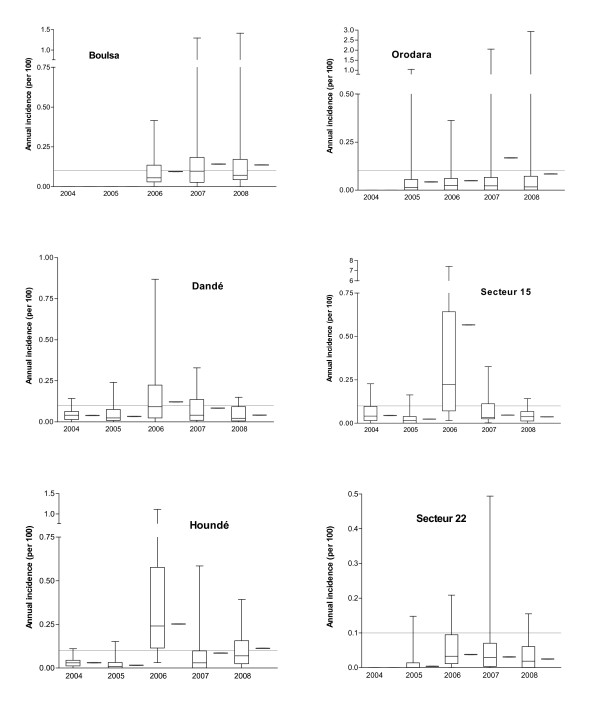
**Annual incidences at health centre and district level in six sanitary districts of Burkina Faso, 2004-2008**. Boxplots show median, 25th and 75th percentile and range of annual incidences at health centre level and red marks show district level annual incidences. Dotted lines indicate annual incidence of 0.1 per 100, previously used as a retrospective definition of epidemics at the district level [7].

A total of 35,078 weekly reports were included. Among the 7,000 reported suspected cases, 93.7% occurred during calendar weeks 1-20, corresponding to the meningitis season with the typical bell shaped incidence curve peaking by end of March and seen every year at district level (Figure 1). During the observed 26 district years, the median annual incidence (5^th ^and 95^th ^percentile) was 0.05% (0.02% and 0.25%) and epidemics at the district level were declared twelve times. Peak WIR (per 100,000) in the six districts were < 10 during 2004 and 2005, 10-60 during 2006, 10-40 during 2007 and 10-30 during 2008. At the health centre level, the highest annual incidence observed was 6.1% and 7.4%, observed in two health centres of the sanitary district Secteur 15 during 2006 (Figure 2).

Annual incidences differed significantly between health centres within the same district year *(P *< 0.001) (Figure 2) and this heterogeneity was greater in district years with official epidemic declaration at the district level: the interquartile range of annual incidences among health centres of the same district year was on median 1.41% (range 0.72-2.29%) in district years with epidemic declaration and 0.03% (0.01-0.09%) in district years without epidemic declaration (Table 1).

**Table 1:** Surveillance of suspected meningitis in six sanitary districts in Burkina Faso, 2004-2008.

		District level				Health center level			
**Year**	**District**	**Population size in 1000 (number of HC)**	**Official epidemic declaration**	**Annual incidence per 100 (case count)**	**Peak WIR**	**HC population size in 1000**	Number of HC with localised epidemics*	**Annual incidence per 100 in HC with localised epidemics †**	**Annual incidence per 100 in HC without localised epidemics †**

2004									
	Dandé	213.3 (21)	No	0.04 (83)	4	9.4 (1.8-22.3)	0	na	na
	Houndé	205.3 (25)	No	0.03 (64)	5	8.4 (3.0-26.3)	0	na	na
	Secteur 15	351.5 (31)	No	0.04 (153)	4	6.7 (1.5-50.3)	0	na	na
2005								na	na
	Dandé	219.7 (21)	No	0.03 (72)	5	9.7 (1.8-23.0)	0	na	na
	Houndé	212.8 (25)	No	0.02 (35)	2	8.7 (3.1-27.2)	0	na	na
	Orodara	244.5 (35)	No	0.04 (106)	4	6.3 (1.1-19.3)	2	0.94 (0.84-1.04)	0.01 (0-0.18)
	Secteur 15	367.7 (32)	No	0.02 (89)	3	6.9 (1.5-53.4)	0	na	na
	Secteur 22	405.9 (21)	No	0.004 (17)	1	12.8 (2.2-76.0)	0	na	na
2006								na	na
	Boulsa	269.1 (30)	Yes	0.10 (255)	12	9.9 (3.5-18.3)	0	na	na
	Dandé	225.6 (22)	Yes	0.12 (275)	23	9.5 (1.9-21.5)	2	0.68 (0.49-0.87)	0.08 (0-0.33)
	Houndé	220.2 (25)	Yes	0.25 (555)	53	9.0 (3.2-28.2)	8	0.60 (0.31-1.11)	0.14 (0.01-0.38)
	Orodara	269.9 (40)	No	0.05 (132)	9	5.9 (1.1-19.9)	0	na	na
	Secteur 15	384.8 (34)	Yes	0.57 (2182)	83	6.1 (1.57-6.6)	10	1.44 (0.32-7.42)	0.13 (0.02-0.49)
	Secteur 22	431.2 (23)	No	0.04 (163)	8	12.7 (2.3-80.5)	0	na	na
2007									
	Boulsa	272.9 (30)	Yes	0.14 (387)	20	10.0 (3.6-18.6)	2	0.91 (0.53-1.30)	0.08 (0.0-1.30)
	Dandé	232.2 (24)	Yes	0.08 (196)	16	9.2 (2.0-22.1)	1	0.33	0.03 (0-0.29)
	Houndé	227.8 (28)	Yes	0.09 (196)	9	7.9 (3.3-18.0)	1	0.59	0.03 (0-0.49)
	Orodara	278.2 (40)	Yes	0.17 (467)	37	6.1 (1.1-20.5)	3	0.45 (0.32-2.05)	0.02 (0-0.45)
	Secteur 15	388.5 (34)	No	0.05 (181)	6	6.3 (1.6-60.0)	0	na	na
	Secteur 22	454.2 (25)	No	0.03 (140)	4	12.9 (2.0-72.7)	0	na	na
2008									
	Boulsa	272.7 (30)	Yes	0.14 (372)	29	10.1 (3.6-18.9)	2	1.12 (0.82-1.41)	0.07 (0.0-1.41)
	Dandé	239.1 (24)	No	0.04 (97)	5	9.5 (2.1-22.8)	0	na	na
	Houndé	235.6 (28)	Yes	0.11 (267)	22	8.2 (3.5-18.7)	0	na	na
	Orodara	286.9 (43)	Yes	0.08 (241)	12	5.64 (1.2-18.7)	1	2.93	0.02 (0-0.32)
	Secteur 15	412.3 (35)	Yes	0.04 (154)	21	6.60 (1.7-63.6)	0	na	na
	Secteur 22	478.6 (25)	No	0.03 (121)	6	13.5 (2.0-77.1)	0	na	na

Population and incidence in 26 surveyed districts years at the district level and at the health centre level. If not indicated otherwise, the health centre level columns show median (range).

Peak WIR, highest weekly incidence rate (per 100,000) observed during a given year

HC, health centers

na, not applicable

* A localised epidemic (LE) was defined at the health centre level as 75 suspected meningitis cases reported per 100,000 inhabitants during at least two consecutive weeks, with a minimum of five cases within at least one week.

† In all district years, the annual incidences between health centres with and without localised epidemic differed significantly (*P *< 0.001, Poisson model).

**Figure 3:**
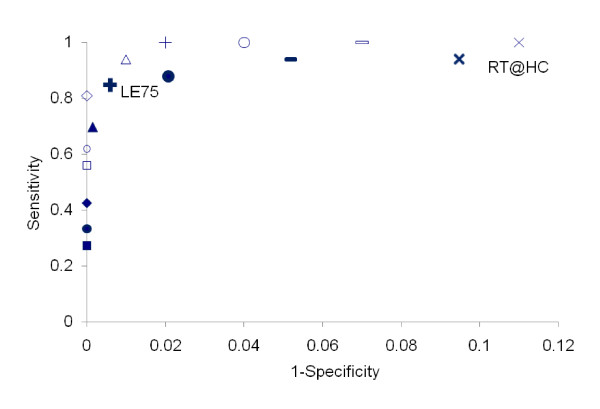
**Performance of localised epidemic definitions in predicting annual meningitis incidences at health centre level**. The different marks represent sensibility and 1-specificity of eight localised epidemic definitions in predicting annual incidences of suspected meningitis at the health centre level in Hauts-Bassins and Centre Nord sanitary region, Burkina Faso, 2004-2008. Full and bold marks represent the performance in predicting annual incidences ≥ 0.4%. Empty or faint marks represent the performance in predicting annual incidences ≥ 0.8%. Definitions for localised epidemics are:- the recommended threshold for epidemic response applied to health centre level data (oblique cross, RT@HC); or - based on different thresholds of weekly incidence rates per 100,000 inhabitants [≥ 25 (bar), ≥ 50 (big circle), ≥ 75 (straight cross, FE75), ≥ 100 (triangle), ≥ 150 (diamond), ≥ 200, (small circles), ≥ 250 (squares)] with incidences maintained during ≥ 2 weeks and with ≥ 5 cases per week.

When the threshold for localised epidemics was varied from LE25 to LE250, sensitivity to identify health centres with annual incidence ≥ 0.4% (≥ 0.8%) varied from 94% to 27% (100% to 56%) and specificity from 95% to 100% (93% to 100%) (Figure 3). RT@HC had a sensitivity of 94% (100%) and a specificity of 91% (89%) in predicting an annual incidence of ≥ 0.4% (≥ 0.8%) with a positive predictive value of 33% (17%) and a negative predictive value of 100% (100%). LE75 had a sensitivity of 85% (100%) and a specificity of 99% (98%) in predicting an annual incidence of ≥ 0.4% (≥ 0.8%) with a positive predictive value of 87% (50%) and a negative predictive value of 99% (100%). We chose this threshold as the definition of localised epidemics based on its robust performance to predict both annual incidence ≥ 0.4% and ≥ 0.8%.

Applying the definition LE75 to the health centre surveillance data, we identified 0, 2, 20, 7 and 3 localised epidemics per year during 2004-2008 (Table 1 and 2). Within district years, annual incidence in health centres with localised epidemics (LE75) were significantly higher than in those without (*P *< 0.001).

**Table 2:** Characteristics of localised epidemics in six sanitary districts, Burkina Faso, 2004-2008.

Year	District	Number of HC with LEs	Calendar week of epidemic declaration at the district level	Calendar week when LE definition was met in the HC (median, range)	Duration of individual LE, median (range) *	Total population in HC with LE, (% of district population)	Predominant agent if laboratory-confirmation during localised epidemic †	Peak WIR in HC with LE, median (range)
2005								
	Orodara	2	no declaration	8 (7-9)	2.5 (2-3)	4,050 (2)	-	318 (169-468)
2006								
	Dandé	2	11	13 (12-14)	2.5 (2-3)	6,850 (3)	28 NmA/31 (*2*)	261 (202-320)
	Houndé	8	9	11.5 (10-14)	3 (2-5)	55,180 (25)	30 NmA/30 (*2*)	191 (115-278)
	Secteur 15	10	5	10 (4-14)	5 (2-12)	97,770 (25)	30 NmA/31 (*1*)	343 (100-1860)
2007								
	Boulsa	2	8	10 (7-13)	6 (2-10)	25,390 (9)	8 NmA/3 Sp/15 (*3*)	190 (93-286)
	Dandé	1	12	14	3 (2-5)	14,290 (6)	-	91
	Houndé	1	12	13	2	5,640 (2)	2 NmA/1 Sp/3 (*1*)	124
	Orodara	3	11	13 (12-14)	3 (2-6)	28,570 (10)	10 NmA/2 Sp/12 (*1*)	104 (94-582)
2008								
	Boulsa	2	10	10.5 (10-11)	5 (4-6)	13,830 (5)	11 NmA/5 Sp/16 (*3*)	276 (185-366)
	Orodara	1	8	8	6	2,490 (1)	4 NmA/4 (*1*)	561

A localised epidemic (LE) was defined at the health centre level as 75 suspected meningitis cases reported per 100,000 inhabitants during at least two consecutive weeks, with a minimum of five cases within at least one week.

WIR, weekly incidence rate

HC, health centres

LE, localised epidemic

NmA, meningococcal serogroup A

* In weeks, includes the week before definition was met

† Number of epidemic agent over number of laboratory-confirmed bacterial meningitis cases

(1) AMP Mobile laboratory [25]

(2) Laboratory surveillance by PCR at Centre Muraz, Bobo-Dioulasso [23]

(3) District level data as reported by Ministry of Health, Epidemiological surveillance department. Burkina Faso

In 9 out of 12 district years with declaration of an epidemic at the district level a localised epidemic was identified in at least one health centre. By contrast, two localised epidemics were identified in one district year without epidemic declaration (Table 1).

**Figure 4:**
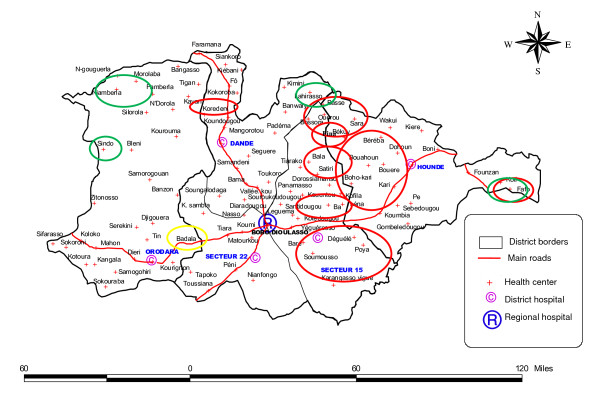
**Localisation of localised epidemics in five districts of the sanitary region Hauts-Bassins, Burkina Faso, 2006-2008**. A localised epidemic (LE) was defined at the health centre level as 75 suspected meningitis cases reported per 100,000 inhabitants during at least two consecutive weeks, with a minimum of five cases within at least one week. Each circle includes the name of one or several health centres identified. Red circles, 2006. Green circles, 2007. Yellow circles, 2008. Map: DRS Hauts-Bassins.

During 2005, two localised epidemics occurred in the western part of the Hauts-Bassins region near the Malian border, with a distance of 100 km to each other. During 2006 in the same region, 17 of the 20 localised epidemics occurred in a geographically contingent zone of about 90 km length and 50 km width which belonged to five different sanitary districts on the current sanitary map, however leaving several health centres within this zone without localised epidemic (Figure 4). In addition, localised epidemics occurred in two neighbouring health centres 70 km to the Nord-West from this cluster and another 60 km to the East. During 2007, five localized epidemics occurred at the borders of the Hauts-Bassins region, one each in the East (the same health centre as in 2006), the North and the West, and two neighbouring health centres in the North-West. During 2008, one single localised epidemic occurred in the South-Western part of the region. In the sanitary district of Boulsa, two localised epidemics occurred during 2007 in the North and South and two in 2008 in the central area.

Localised epidemics occurred in small populations: the median size of concerned health centre populations was 6,702 (range 1,068-22,472) corresponding in median to 6% (range 1%-25%) of the district population (Table 2). The criteria for localised epidemics were fulfilled in median during calendar week 10 (range 3-13) and epidemics lasted for 3 weeks (2-12). Where sufficient laboratory data were available, localised epidemics were exclusively due to meningococci (Table 2). Within district years, the district annual incidence correlated positively with the number of localised epidemics identified in the district (rho = 0.829, *P *= 0.003), the total population referring to health centres concerned by localised epidemics (rho = 0.878, *P *< 0.001) and the percentage of district population concerned (rho = 0.859, *P *= 0.002), but not with the median annual incidence observed in health centre years with localised epidemics (rho = 0.024, *P *= 0.947), median peak WIR (rho = -0.031 *P *= 0.931) or median duration of localised epidemics (rho = 0.290, *P *= 0.417).

Districts tended to declare epidemics earlier than localised epidemics could have been identified by LE75 (Table 2). Using RT@HC, 95 localised epidemics were detected, 40 after epidemic declaration in the district, 20 without occurrence of an epidemic declaration in the district and 35 with consecutive epidemic declaration in the district (yielding a PPV of 63% for epidemic detection by RT@HC compared to district declaration). In 20 of the 75 health centres (27%) situated in districts that declared an epidemic during a given year, a localised epidemic was identified by RT@HC at least one week earlier than the district declared the epidemic.

## Discussion

This evaluation is the first attempt to quantify and characterize localised meningitis epidemics in the meningitis belt at the health centre level. The advantage of our definition in comparison to existing district level approaches [18,19] is that it allows attributing an epidemic event precisely to specific communities and the exact calendar weeks, while the analysis is based on national case reporting systems which collect data routinely in virtually all sanitary districts of the meningitis belt and are available for several past years.

By applying a definition of ≥ 75 cases per 100,000 inhabitants during at least two weeks and ≥ 5 cases during at least one of these weeks to these health centre level data, we could describe several characteristics of meningitis epidemiology in general, and localised epidemics in particular. First, we observed great heterogeneity in weekly and annual incidence among health centres of the same district, especially during epidemic years. This confirms that, to fully understand the dynamic of meningitis epidemics, health centre level data should be analysed.

Secondly, in most years, localised epidemics concerned small communities (< 5,000 inhabitants in several instances), small portions of the district population (as low as 1%), restricted geographic areas (individual health centre zones) and short periods (as short as two weeks). These elements together point to the fact that epidemics at the community level are events that are distinct from ubiquitous hyper-endemic meningitis incidence during the dry season, which is most likely driven by climatic factors [19,20], and distinct also from epidemic waves across larger regions, which occur every 10 years or so [21]. While no strong evidence exists on what makes these localised epidemics occur, specific spatially restricted factors other than climate or meningococcal strains are likely responsible, possibly including micro-epidemics of viral infections [13].

Third, the importance of the epidemic at the district level correlated with the number of localised epidemics in the district, but not with the duration or the level of incidence rates during the localised epidemics. If epidemics at the district level are considered the smallest form of epidemic waves, our results lend support to the concept that epidemic waves are the sum of individual localised epidemics and their importance depend on the number of localised epidemics that occur simultaneously. In consequence, the occurrence of epidemic waves would be triggered by a greater than usual geographic expansion of local factors that drive the epidemic, rather than the quality or intensity of a distinct cause of epidemic waves.

Fourth, localised epidemics with appropriate laboratory evaluation appeared to be caused by meningococcus and not by pneumococcus, although the latter was occasionally identified. Despite the fact that pneumococci are responsible for almost half of bacterial meningitis cases in the meningitis belt [22,23] and cause meningitis with pronounced seasonality, no localised epidemic so far has been reported due to pneumococci, which suggests that the focal epidemic pattern is limited to meningococci. Serogroups W135 or X were found only sporadically in Burkina Faso during the observed years, such that we cannot evaluate whether these serogroups cause localised epidemics, as well. An analysis of the recent years with high pneumococcal contribution to seasonal meningitis and the NmX epidemic in Burkina Faso during 2010 [5] should evaluate these hypotheses. Because only a relatively small proportion of suspected cases actually are meningococcal meningitis cases, as some studies have shown [4,22,24], epidemiological analyses of these surveillance data can yield evidence with only limited precision. If the explosive character of localised epidemics was typical for meningococci, the proposed definition could help overcome this limitation of routine surveillance data and allow analyses that are specific for meningococcus.

Lastly, localised epidemics occurred in 38% of the 26 evaluated district years, while in the total evaluated population (1.9 million), at least two epidemics were identified during each year, except 2004. That year showed moderate meningitis incidence in Burkina Faso and it is likely that localised epidemics occurred elsewhere in the country. We hypothesize that in a somewhat larger population than that evaluated here (e. g., three sanitary regions), several localised epidemics occur every year, even outside epidemic waves. The disappearance of these localised epidemics could be used for quantifying the impact that meningococcal serogroup A conjugate vaccine (MenAfriVac^®^) will have on epidemic meningitis. The introduction of MenAfriVac^® ^in the meningitis belt is expected to substantially change the meningitis epidemiology and a key issue will be to evaluate the impact of the vaccine on the frequency and extend of epidemics. It is possible that the presented definition of epidemics at the health centre level will allow quantifying the long-term impact in a sensitive and economic way, especially in areas where no widespread laboratory surveillance is conducted.

In three instances, an epidemic was declared at district level although the proposed definition LE75 did not identify any localised epidemic. Detailed analyses showed that typically a few health centres had WIR above 75 per 100,000 but only during one week or with < 5 cases per week. The proposed definition appears to have a reduced sensitivity in these instances, but for the sake of robustness and specificity of the definition, as needed for risk factor research, we preferred to maintain the requirement of two consecutive weeks with high WIR.

While the proposed definition of localised epidemics at the health centre level (LE75) did not improve timeliness of epidemic declaration at the health centre level compared to the current practice of district level analysis, our study showed that if RT@HC was used, epidemics could be identified geographically precisely and in one quarter of instances at least one week earlier than by district level analysis. This may be important for reactive mass vaccine campaigns, which will remain necessary after MenAfriVac^® ^introduction, at least for occasional serogroup W135 epidemics [2,3]. Some countries, such as Mali and Togo, do organize reactive vaccine campaigns based on health centre level incidence data and therefore hold vaccine stocks at district level. Many factors influence the effectiveness and costs of a reactive vaccination campaign (speed of data transmission, campaign logistics, and target population) and the usefulness of health centre level analysis of surveillance data for informing vaccination strategies need to be evaluated in more detail on larger data sets.

Our study has several limitations. We used data from a surveillance system without routine quality control and some biases may arise. For example, heterogeneity between health centre level incidences may result from differences in reporting practices. Incidences during the rainy season were comparable in health centres across all districts, but differences in practices may arise specifically during the meningitis season. We included only a small sample of districts spanning five years and there may be geographic variations in the nature of localised epidemics. However, our study included two years outside epidemic waves (2004-5) and three years of an epidemic wave (2006-8), and therefore could be representative of the longer-term variations in epidemiology in Burkina Faso. Data on past vaccination campaigns and coverage at the health centre level were not available. Population immunity likely is an important factor of epidemic occurrence and heterogeneous vaccination coverage across the district may explain why some outbreaks remain localised and why outbreaks may occur several years in a row despite vaccination campaigns.

We excluded cases reported from reference medical centres as they could not be assigned to a specific health care centre population. This certainly led to an underestimation of incidence rates, especially in health centres close to reference centres. Similarly, patients infected in other health centres, districts or regions may have been included in a health centre case report due to consultation preference or travel. However, we believe that our definition of localised epidemics is sufficiently robust not to be biased by this, except in the case of major population movements.

To validate the approach, similar analyses should be conducted on a wider geographical area and include more recent years that were characterized by low serogroup A incidence and high serogroup X or pneumococcal incidence, use spatial epidemiological methods and include more systematic laboratory information on the aetiology of meningitis epidemics and vaccination. Also, it will be interesting to evaluate at which interval localised epidemics occur in individual communities.

## Conclusion

In summary, this evaluation of health centre level data confirmed that localised epidemics are events that are distinct from hyperendemicity or regional epidemic waves. Further research should identify the factors that lead to transition from hyperendemic meningitis incidence during the dry season to localised epidemics, and from individual localised epidemics to larger epidemic waves. The definition we proposed and used to describe characteristics of localised epidemics should be validated on larger data sets and may be useful for future risk factor and modelling studies and for assessment of vaccine impact on epidemic meningitis in the African meningitis belt.

## Competing interests

HT and JEM work for AMP, which receives unrestricted financial support from Sanofi Pasteur. SH, PD, MDW, LK and FD declare that they have no competing interests.

## Authors' contributions

HT, JEM, PD and MDW designed the study, HT, SH, LK and FD contributed to data collection, HT managed and analyzed the data, HT and JEM wrote the manuscript, and all authors contributed to data interpretation and manuscript revision, and read and approved the final draft.

## Authors' information

SH is a staff member of the World Health Organization. The author alone is responsible for the views expressed in this publication and they do not necessarily represent the decisions, policy or views of the World Health Organization.

## Pre-publication history

The pre-publication history for this paper can be accessed here:

http://www.biomedcentral.com/1471-2334/12/2/prepub

## References

[B1] Stephens DS Greenwood B Brandtzaeg P Epidemic meningitis, meningococcaemia, and *Neisseria meningitidis* Lancet 2007 369 2196 2210 10.1016/S0140-6736(07)61016-2 17604802

[B2] Mueller JE Borrow R Gessner BD Meningococcal serogroup W135 in the African meningitis belt: epidemiology, immunity and vaccines Expert Rev Vaccines 2006 5 3 10.1586/14760584.5.3.319 16827617

[B3] Collard JM Maman Z Yacouba H Djibo S Nicolas P Jusot JF Rocourt J Maitournam R Increase in *Neisseria meningitidis *Serogroup W135, Niger, 2010 Emerg Infect Dis 2010 16 1496 1498 20735947 10.3201/eid1609.100510 PMC3294991

[B4] Boisier P Nicolas P Djibo S Taha MK Jeanne I Maïnassara HB Tenebray B Kairo KK Giorgini D Chanteau S Meningococcal meningitis: unprecedented incidence of serogroup X-related cases in 2006 in Niger Clin Infect Dis 2007 44 657 663 10.1086/511646 17278055

[B5] Delrieu I Yaro S Tamekloé TA Njanpop-Lafourcade BM Tall H Jaillard P Ouedraogo MS Badziklou K Sanou O Drabo A Gessner BD Kambou JL Mueller JE Emergence of epidemic Neisseria meningitidis serogroup X meningitis in Togo and Burkina Faso PLoS One 2011 6 5 e19513 Epub 2011 May 20 10.1371/journal.pone.0019513 21625480 PMC3098835

[B6] World Health Organization Detecting meningococcal meningitis epidemics in highly-endemic African countries Weekly Epidemiological Record 2000 75 305 312 11045076

[B7] Lewis R Nathan N Diarra L Belanger F Paquet C Timely detection of meningococcal meningitis epidemics in Africa Lancet 2001 358 287 293 10.1016/S0140-6736(01)05484-8 11498215

[B8] World Health Organization Enhanced surveillance of epidemic meningococcal meningitis in Africa: a three-year experience Weekly Epidemiological Record 2005 80 313 320 16193931

[B9] Thomson MC Molesworth AM Djingarey MH Yameogo KR Belanger F Cuevas LE Potential of environmental models to predict meningitis epidemics in Africa Trop Med Internat Health 2006 11 78781 78788 10.1111/j.1365-3156.2006.01630.x 16771998

[B10] Greenwood BM Bradley AK Cleland PG Haggie MH Hassan-King M Lewis LS Macfarlane JT Taqi A Whittle HC Bradley-Moore AM Ansari Q An epidemic meningococcal infection at Zaria, Northern Nigeria. 1. General epidemiological features Transact Royal Soc Trop Med Hygiene 1979 73 557 562 10.1016/0035-9203(79)90052-X 531909

[B11] Sié A Pflüger V Coulibaly B Dangy JP Kapaun A Junghanss T Pluschke G Leimkugel J ST-2859 serogroup A meningococcal meningitis outbreak in Nouna Health District, Burkina Faso: a prospective study Trop Med Internat Health 2008 13 861 868 10.1111/j.1365-3156.2008.02056.x 18384478

[B12] Mueller JE Yaro S Njanpop-Lafourcade BM Drabo A Idohou RS Kroman SS Sanou O Diagbouga S Traoré Y Sangaré L Borrow R Gessner BD Study of a localized meningococcal meningitis epidemic in Burkina Faso: incidence, carriage and immunity J Infect Dis 2011 204 1787 1795 10.1093/infdis/jir623 21998478 PMC3247801

[B13] Mueller JE Gessner BD A hypothetical explanatory model for meningococcal meningitis in the African meningitis belt Int J Infect Dis 2010 15 553 559 10.1016/j.ijid.2009.08.013 20018546

[B14] Traore Y Tamekloe TA Njanpop-Lafourcade BM Lourd M Yaro S Niamba D Drabo A Mueller JE Koeck JL Gessner BD Pneumococcal meningitis in Burkina Faso and Togo is common, seasonal, affects all age groups, and is highly lethal Clin Infect Dis 2009 48 S181 189 10.1086/596498 19191614

[B15] World Health Organization WHO Recommended Strategies for the Prevention and Control of Communicable Diseases http://whqlibdoc.who.int/hq/2001/WHO_CDS_CPE_SMT_2001.13.pdf

[B16] United States Agency for International Development Burkina Faso. Water and sanitation profile http://pdf.usaid.gov/pdf_docs/PNADO927.pdf

[B17] Ministère de la Santé Burkina Faso Annuaire Statistique Santé 2007 http://www.sante.gov.bf/SiteSante/statistiques/annuaire-2007.pdf

[B18] Leake JAD Kone ML Yada A Barry LF Traore G Ware A Coulibaly T Berthe A Mambu Ma Disu H Rosenstein NE Plikaytis BD Esteves K Kawamata J Wenger JD Heymann DL Perkins BA Early detection and response to meningococcal disease epidemics in sub-Saharan Africa: appraisal of the WHO strategy Bull WHO 2002 80 342 349 12077608 PMC2567794

[B19] Moore PS Meningococcal meningitis in sub-Saharan Africa: a model for the epidemic process Clin Infect Dis 1992 14 515 525 10.1093/clinids/14.2.515 1554841

[B20] Sultan B Labadi K Guégan JF Fanicot S Climate drives the meningitis epidemics onset in West Africa PLoS Medicine 2005 2 43 49 10.1371/journal.pmed.0020043 PMC545199 15696216

[B21] World Health Organization Risk of epidemic meningitis in Africa: a cause for concern Weekly Epidemiological Record 2007 10 79 87

[B22] Parent du Châtelet I Traore Y Gessner BD Antignac A Naccro B Njanpop-Lafourcade BM Ouedraogo MS Tiendrebeogo SR Varon E Taha MK Bacterial meningitis in Burkina Faso: surveillance using field-based polymerase chain reaction testing Clin Infect Dis 2005 40 17 25 10.1086/426436 15614687

[B23] Yaro S Lourd M Traoré Y Njanpop-Lafourcade BM Hien A Ouedraogo MS Traore Y Schouls LM Parent du Châtelet I Gessner BD Clinical Group; Laboratory Group Epidemiological and molecular characteristics of a highly lethal pneumococcal meningitis epidemic in Burkina Faso Clin Infect Dis 2006 43 693 700 10.1086/506940 16912941

[B24] Rose AMC Mueller JE Gerstl S Njanpop-Lafourcade BM Page AL Nicolas P Traoré RO Caugant DA Guerin PJ Meningitis dipstick rapid test: evaluating diagnostic performance during an urban *Neisseria meningitidis *serogroup A outbreak, Burkina Faso, 2007 PLoS ONE 2010 5 e11086 10.1371/journal.pone.0011086 20552035 PMC2884039

[B25] Ouedraogo RT Njanpop-Lafourcade BM Jaillard P Traoré Y Mueller JE Aguilera JF Dabal M Tiendrébéogo SR Goehde W Da Silva A Gessner BD Stoeckel P Mobile laboratory to improve response to meningitis epidemics, Burkina Faso epidemic season 2004 http://factsreports.revues.org/144 Accessed 18 Novembre, 2011

